# Supplementary figures and images for: Effect of heat-killed Streptococcus thermophilus on type 2 diabetes rats
Source: PeerJ. 2019 Jun 13;7:e7117. doi: 10.7717/peerj.7117 (PMC6571132; doi:10.7717/peerj.7117)

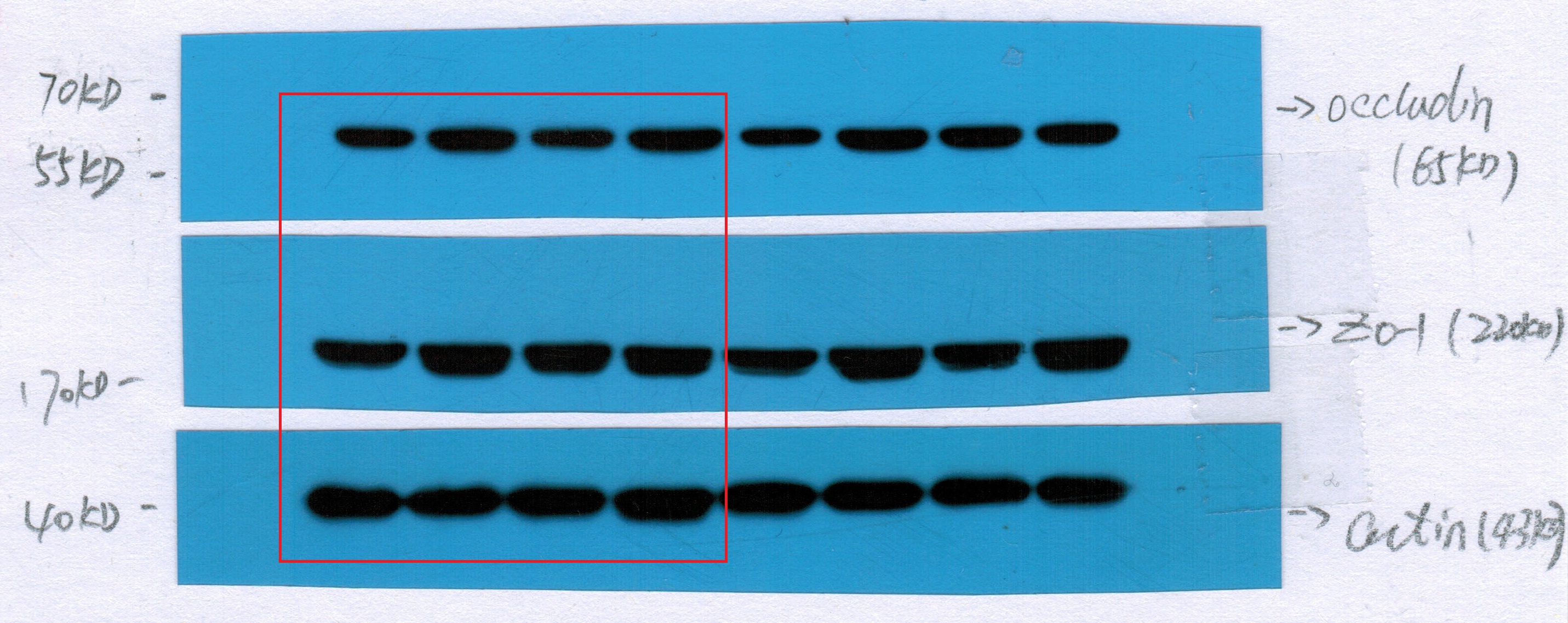

Supplement: Supplemental Information 2 [file peerj-07-7117-s002.jpg]

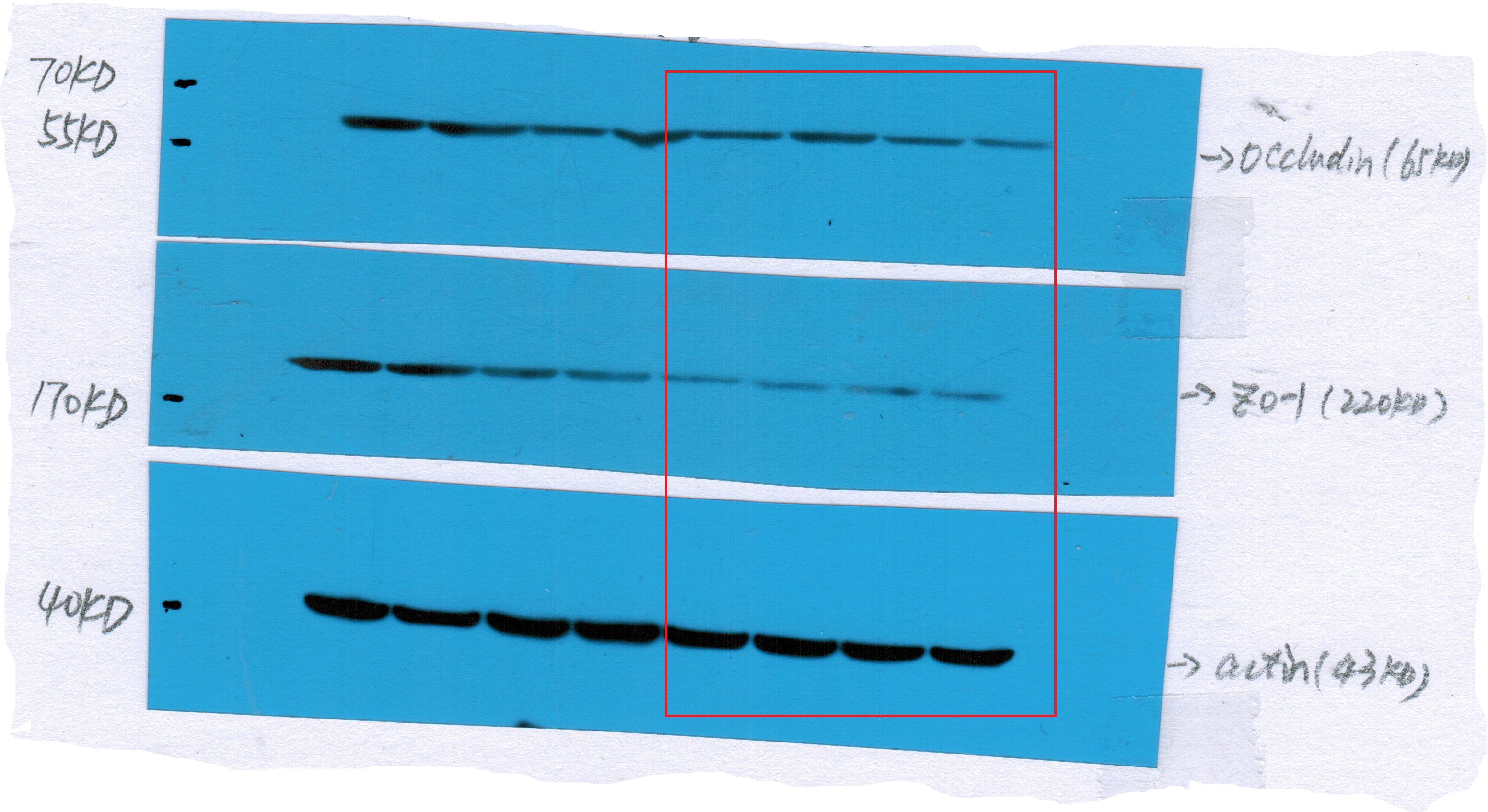

Supplement: Supplemental Information 3 [file peerj-07-7117-s003.jpg]
